# Supplementary material for: Prediction of Metabolic Profiles from Transcriptomics Data in Human Cancer Cell Lines
Source: Int J Mol Sci. 2022 Mar 31;23(7):3867. doi: 10.3390/ijms23073867 (PMC8998886; doi:10.3390/ijms23073867)
Supplement: Supplementary file 1 [file ijms-23-03867-s001.zip › Figure S1.pdf]

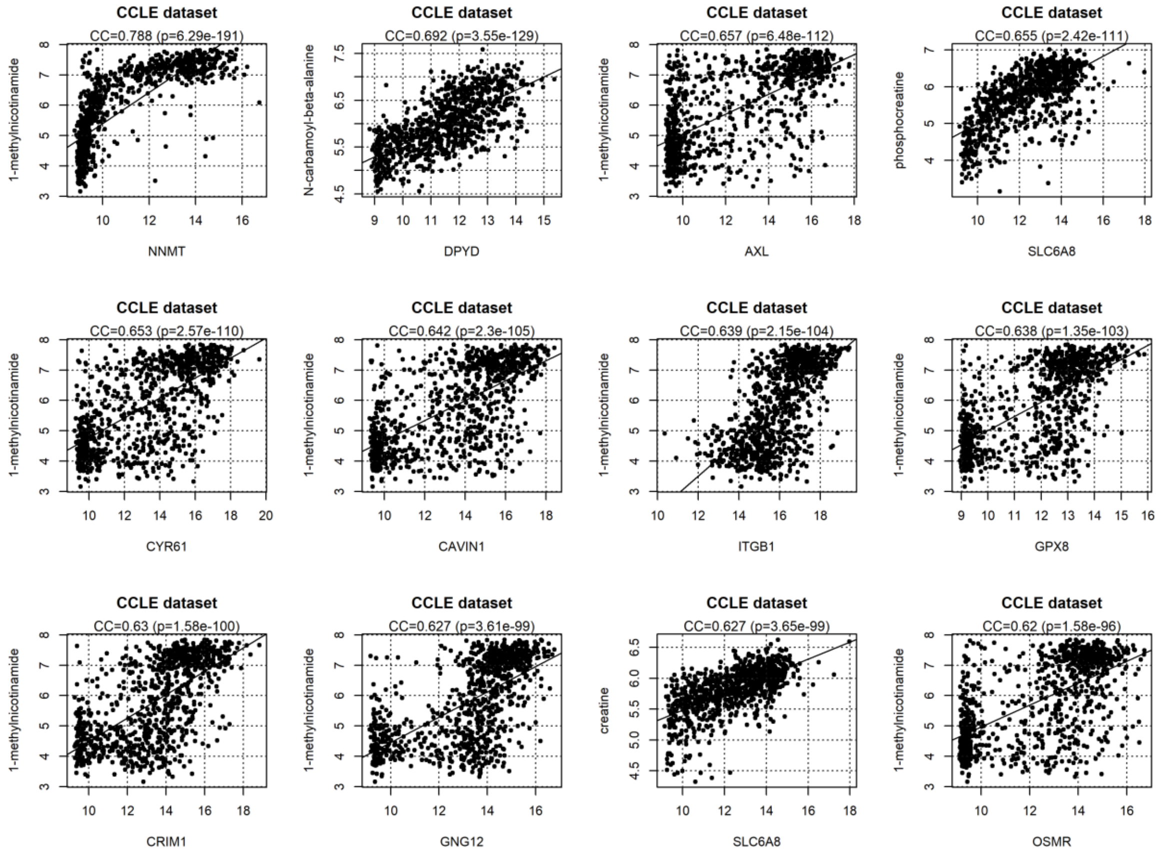

Figure S1: correlation between metabolite levels and gene expression in the CCLE dataset, showing the 12 pairs with the highest correlation coefficient.
